# Unusual indication of Cell therapy for hip osteonecrosis after pregnancy

**DOI:** 10.1051/sicotj/2018044

**Published:** 2018-11-05

**Authors:** Philippe Hernigou, Guillaume Rigoulot, Jean Charles Auregan, Victor Housset, Claire Bastard, Arnaud Dubory, Charles Henri Flouzat Lachaniette

**Affiliations:** 1 Hôpital Henri Mondor, 51 avenue du Maréchal de Tassigny, 94000 Creteil France; 2 Auregan Jean Charles, Hopital Antoine Beclere, 157 Rue de la Porte de Trivaux, 92140 Clamart France

**Keywords:** Cell therapy, Mesenchymal stem cell, Hip osteonecrosis, Pregnancy

## Abstract

*Purpose*: Osteonecrosis is a rare event during or after pregnancy. Due to the low number of cases, there is no report of long-term results after conservative surgery in this population.

*Method*: From 1992 to 2010, 145 consecutive female patients with unilateral symptomatic (94 stage I and 51 stage II) left hip osteonecrosis (ON) related to pregnancy were treated between 4 and 16 months after delivery with percutaneous mesenchymal stem cell (MSC) injection obtained from bone marrow concentration. The average total number of MSCs (counted as number of colonies forming units-fibroblast) injected in the hip was 185 000 ± 65 000 cells (range 95 000–240 000 cells).

*Results*: At the most recent follow-up (average 15 years after hip surgery, range 8–25 years), among the 145 hips included in the study, 4 hips (2.7%) had collapsed and were treated with total hip arthroplasty (THA). Thirty-two of the 94 stage I had progressed to stage II; and 4 of the 51 stage II had progressed to stage III and collapse. The other 141 hips (97.3%) were without collapse and pain free. On MRI, as percentage of the volume of the femoral head, the osteonecrosis had decreased from 34.5% pre-operatively to 6% at the most recent follow-up. For women who had other children after treatment, a subsequent pregnancy was not associated with osteonecrosis.

*Conclusion*: Patients with hip osteonecrosis following pregnancy had a low conversion rate to THA when treated early with cell therapy.

## Introduction

In a cohort-crossover analysis of a large sample of healthy women with osteonecrosis, we found association between pregnancy and the risk of osteonecrosis confirming a relationship between hip osteonecrosis and pregnancy [1]. Women without documentation of established risk factors for osteonecrosis appear to have an increased relative risk of osteonecrosis during pregnancy; the absolute risk may be considered as low as regards to a complication of pregnancy, but is not insignificant according to the percentage of cause of osteonecrosis in our data. With a roughly analysis, we can remark that for the diagnosis of osteonecrosis related to a pregnancy, we observed during a long period (from 1985 to 2010) 288 pregnant osteonecrosis (6%) among the 4746 women with osteonecrosis related to another cause. As the number of osteonecrosis is around 5000 for women each year in our country, this means that probably the risk is around 300 new cases every year; the population is around 60 million people with 800 000 pregnancies each year [2]; so, the risk can be estimated around 0.375 per 1000 pregnancies and is very low.

In this circumstance of pregnancy, osteonecrosis affects very young patients, and it was traditionally believed that any procedure able to preserve the patient's native hip is preferable in young patients. Even if this enthusiasm for conservative treatment is now tempered with our acknowledgment of time-proven hip arthroplasty [3,4] as a reasonable surgical alternative for collapsed hips, the conservative treatment (and particularly without change of the morphology of the hip) remains the best treatment for young patients when it is able to prevent collapse.

We therefore proposed to this population of young women as treatment of osteonecrosis a percutaneous mesenchymal stem cell (MSC) injection obtained from bone marrow concentration [5,6]. If we accept pregnancy as cause of ON, whatever the mechanism of the ON, the situation is normalised after delivery: the patient should have normal number and function of MSCs in the iliac wing and the femoral head (except the osteonecrosis) is a normal bone contrary to what can be observed with other cause of osteonecrosis [7,8]; the femoral head is a “diseased” bone due to the cause of ON (corticoids, alcohol abuse...). These women with osteonecrosis related to pregnancy, therefore, should be the best indication for this treatment.

With this hypothesis, we report here the mean 15-year follow-up of a cohort of 145 consecutive female patients with unilateral symptomatic hip osteonecrosis (ON) related to pregnancy who were treated from 1992 to 2010 with a percutaneous MSCs injection obtained from bone marrow concentration. The efficacy of this autologous implantation is analyzed in this situation and compared with the results of other series in the discussion.

## Material and methods

### Patients' demography

Among all the women who were treated for osteonecrosis at our institution, we analyzed the evolution of 145 patients who were healthy (without any known cause of osteonecrosis) before pregnancy, who had an osteonecrosis during pregnancy, or 1 year after labor, and who were treated with cell therapy. These patients had a mean age of 26 years (range 18–34 years) at the time of the onset of symptoms. Hips with collapse (stage III or IV osteonecrosis) of the femoral head were not included. Only early symptomatic osteonecrosis (stage I or II as defined by Steinberg [9], or ARCO classification [10]) was included.

Patients with known associations as corticosteroids treatment, sickle cell disease, hyperlipidemia, alcoholism, Cushing's syndrome, lupus, rheumatoid arthritis, etc., were excluded to avoid confounding factors and bias. Bilateral hip osteonecrosis was excluded to avoid the risk of unknown disease as a cause. As in the literature (theory of excessive compression of the iliac veins on the left side from the weight of the developing fetus) and according to our experience left hip is more frequently involved when osteonecrosis is related to pregnancy; therefore, we only kept left hips for this study.

The diagnosis of osteonecrosis was made on MRI with the basis of a finding of a double density signal on magnetic resonance imaging. The diagnosis of transient osteoporosis was excluded on the basis of MRI, and of the clinical course, as none of these patients had spontaneous remission on the magnetic resonance imaging.

All patients were followed up within our institution with a clinical examination and radiographs every year through 2018. Surgery was performed between 1992 and 2010. The follow-up is average 15 years (range 8–25 years). Clinical progression was defined as progression of pain (i.e. symptoms). Radiographic progression was defined as an advance in the Steinberg stage or progressive collapse. Changes in lesion size of lesion were determined on different MRIs at the preoperative period and at the most recent follow-up.

### Operative technique

This technique has been reported several times [11,12]. Briefly, under general anaesthesia patients the trocar was introduced percutaneously through the trochanter. The position of this 4 mm diameter trocar was monitored in the femoral head with C-arm fluoroscopy. An average of 150 mL of marrow was aspirated from the iliac wing. After concentration, the bone marrow was injected into the femoral head. Patients were allowed immediate weight bearing using crutches for 10 days; full weight bearing without crutches was allowed after 10 days.

### Cell analysis

As previously reported [13], we used the colony forming unit fibroblast (CFU-F) as an indicator of stromal cell activity to measure bone marrow MSCs number. In summary, two parameters were calculated from cell culture; by counting bone marrow smears on a hemocytometer, the bone marrow mono-nucleated-cell count was evaluated as the number of bone marrow mononucleated cells per 1 cc of marrow aspirate; this number ranged from 26 to 40 million per milliliter with a mean value of 38 ± 7 million per milliliter. The frequency of MSCs per 1 million mononucleated cells was estimated by counting the number of CFU-Fs present in culture. After 10 days in culture, an average of 48.6 ± 19.9 CFU-F colonies per million of mononucleated cells was observed in BM aspirates cultures. Each femoral head received 20 mL of concentrated (5 times) bone marrow whatever the size of the necrotic lesion. The average total number of MSCs (counted as CFU-F) injected in each hip was 185 000 ± 65 000 cells (range 95 000–240 000 cells).

### Statistical analyses

Data are reported as mean ± standard deviations and the significance level was set at a probability value less than 0.05. The outcome variables were the treatment success (absence of collapse, absence of THA), the clinical and radiographic change of the disease (pain, stage, and size change), and the volumetric evaluation of the repair tissue (one MRI) after bone marrow transfer. The local factors that were considered able to influence the outcome variables were the total number and the concentration of MSCs injected in the osteonecrosis, the volume of osteonecrosis, and the stage of ON. Correlations between the outcome variables and the cells factors were determined using Spearman's correlation test. The non-parametric Mann–Whitney *U* test was used to compare the differences between the different groups. The Chi-square test was used to identify trends within groups with categorical variables.

## Results

### Bone marrow grafting improved pain and joint symptoms

Overall, whatever size and stage of osteonecrosis, pain and Harris scores were improved at the end of the first month. The mean Harris hip score progressed significantly from 76 points (range, 65–82 points) before surgery to 94 points (range, 85–100 points) at 3 months post-surgery (*p* < 0.01). The mean VAS score (pain score in millimeters) reduced from 41.2 ± 6.5 before surgery to 12 ± 3.5 at 3 months post-intervention (*p* < 0.01), and to 6 ± 4 at 12 months.

Recurrence of pain occurred in 12 hips. When recurrence of pain occurred, there was a risk of collapse since during the first 5 years of our treatment 4 collapse was observed. However, recurrence of pain was always observed before collapse for these hips that progressed towards collapse. Therefore, for the other 8 hips that presented recurrence of pain during follow-up, a repeat bone marrow injection was performed after a new MRI demonstrating persistence of the osteonecrosis; these 8 patients returned as a pain free level and had no collapse.

### Bone marrow graft improved the repair process on MRI

#### Repair process was observed on MRI

The pre-operative average volume of ON ranged was 18.3 cc (range 41–11 cc) corresponding to a mean value of 34.5% as percentage of the volume of the femoral. 55 hips (initially stage I) showed a total regression of their abnormal signal on MRI, 72 hips showed a partial (>10%) reduction (no collapse) of the signal while 18 hips did not show a significant (<10%) reduction (12 had recurrent pain and 4 collapse). Overall on MRI, as percentage of the volume of the femoral head, the osteonecrosis had decreased from 34.5% pre-operatively to 6% at the most recent follow-up.

#### For hip repair, volume of osteonecrosis, stage and number of cells were influent factors

There was a positive correlation between the volumes of repair obtained at the most recent follow-up and the concentration of MSCs in the graft (*p* = 0.001). The larger lesions and the stage II lesions (compared to stage I lesions) had a significant lower volume of repair and a lower rate of repair for similar matched dose of MSCs. At the most recent follow-up 32 of the 94 stage I had progressed to stage II; and 4 of the 51 stage II had progressed to stage III and collapse.

#### Cartilage wear and osteoarthritis was not observed in long term

Cartilage thickness was evaluated on radiographs and on MRI. Hips treated with bone marrow implantation had normal thickness of the cartilage, except 6 among the 141 hips without collapse. MRI did not show changes in the cartilage signal for the other hips.

### Failure and conversion rate to THA was low

At the most recent follow-up (average 15 years after hip surgery, range 8–25 years), among the 145 hips included in the study, 4 hips (2.7%) had collapsed and were treated with total hip arthroplasty (THA). These women had another normal pregnancy without osteonecrosis on the contralateral hip and a normal vaginal delivery. Precautions during vaginal delivery included limitation of internal rotation to maintain the joint in neutral position.

### A subsequent pregnancy was not a risk of hip osteonecrosis

For the 96 women who had other children after treatment, a subsequent pregnancy was not associated with osteonecrosis on the hip that was treated or on the contra-lateral hip. For these women as for those who had not a second pregnancy, no disease that could have been a cause of osteonecrosis was observed.

## Discussion

When hip pain occurs in a pregnant woman [14], it is important to distinguish osteonecrosis from bone marrow edema syndrome (or algodystrophy), a condition known to occur with greater frequency in pregnant women. Bone marrow edema syndrome is distinguished from ON because osteonecrosis has a characteristic serpiginous border between viable and nonviable bone seen on T1- and T2-weighted MRI images. Bone marrow edema syndrome will not have this distinct serpiginous border and, contrary to ON, will have abnormal bone marrow signal extending in the femoral neck and into the trochanteric region. Furthermore, the MRI changes seen in bone marrow edema syndrome reverse to normal, whereas those due to ON persist with time.

It is well established that untreated osteonecrosis progresses to femoral head collapse with subsequent hip osteoarthritis in most of the cases. It remains difficult for the orthopedic surgeon to feel justified a THA even with new implants [15,16] in a young patient simply for hip pain without femoral head collapse. Several conservative approaches [17,18] have been proposed for osteonecrosis treatment including weight restriction and observation, core decompression, various osteotomies, and bone grafting (structural or nonstructural and fibula vascularized or non-vascularized). However, these techniques have high failures reports for some of them, and others change the morphology of the hip which may be non-esthetic in a young woman [19]. Our technique demonstrates good results and better results than those observed in other causes of osteonecrosis with the same technique [12,20,21]. This is probably in relation with the fact that pregnancy is not a disease and that after delivery the situation is normalised. This explains the high level of MSCs observed in the iliac wing as compared with patients developing osteonecrosis related to corticosteroid treatment [12,22]. This also probably explains the high rate of repair in the hip after such a treatment.

The findings of this study must be interpreted in the context of several limitations. First, no hip MRI was performed before pregnancy or osteonecrosis diagnosis; therefore, our findings may reflect some previous undiagnosed or undocumented idiopathic osteonecrosis with aggravation during pregnancy and not only osteonecrosis related to pregnancy. This may limit the number of hip osteonecrosis related to pregnancy, but does not limit the results of cell therapy in osteonecrosis… Second, according to the large number of causes of osteonecrosis reported in the literature, some cause of osteonecrosis might be undiagnosed in our population resulting in misclassification.

Our finding has implications for pre-pregnancy counseling and peri-partum care. A scarcity of data [23–27] exists to guide women with the risk of osteonecrosis for primary osteonecrosis or for the risk of a second osteonecrosis for a second pregnancy. We have probably the larger series of osteonecrosis relate to pregnancy. According to our data, no osteonecrosis was observed during a subsequent pregnancy, which is logical according to a risk that we evaluated as less than 1/1000. The same remark can be done for healthy women who had osteonecrosis before a gestation.

## Conflict of interest

The authors declare that they have no conflicts of interest in relation to this article.
